# Methyl 2-methyl-2*H*-1,2,3-triazole-4-carboxyl­ate

**DOI:** 10.1107/S1600536809024829

**Published:** 2009-07-04

**Authors:** K. Prabakaran, Venkatesha R. Hathwar, T. Maiyalagan, M. V. Kirthana, F. Nawaz Khan

**Affiliations:** a Chemistry Division, School of Science and Humanities, VIT University, Vellore 632 014, Tamil Nadu, India; b Solid State and Structural Chemistry Unit, Indian Institute of Science, Bangalore 560 012, Karnataka, India

## Abstract

In the title compound, C_5_H_7_N_3_O_2_, all non-H atoms lie in a common plane, with a maximum deviation of 0.061 (2)° for the ester methyl C atom. The structure is stabilized by inter­molecular C—H⋯O hydrogen bonds.

## Related literature

For general background to the applications of triazoles and their derivatives, see: Abu-Orabi *et al.* (1989 ▶); Fan & Katritzky (1996 ▶); Dehne (1994 ▶); Wang *et al.* (1998 ▶). For a related structure, see: Prabakaran *et al.* (2009 ▶).
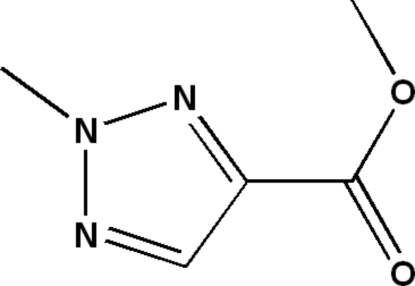


## Experimental

### 

#### Crystal data

C_5_H_7_N_3_O_2_
*M* *_r_* = 141.14
Monoclinic, 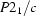

*a* = 3.9482 (10) Å
*b* = 7.9549 (15) Å
*c* = 21.655 (4) Å
β = 92.05 (2)°
*V* = 679.7 (2) Å^3^
*Z* = 4
Mo *K*α radiation
μ = 0.11 mm^−1^
*T* = 290 K
0.30 × 0.20 × 0.10 mm

#### Data collection

Oxford Xcalibur Eos(Nova) CCD detector diffractometer
Absorption correction: multi-scan (*CrysAlisPro RED*; Oxford Diffraction, 2009 ▶) *T*_min_ = 0.926, *T*_max_ = 0.989
7464 measured reflections
1262 independent reflections
910 reflections with *I* > 2σ(*I*)
*R*_int_ = 0.043

#### Refinement

*R*[*F* ^2^ > 2σ(*F* ^2^)] = 0.042
*wR*(*F* ^2^) = 0.119
*S* = 1.07
1262 reflections
93 parameters
H-atom parameters constrained
Δρ_max_ = 0.19 e Å^−3^
Δρ_min_ = −0.17 e Å^−3^

### 

Data collection: *CrysAlisPro CCD* (Oxford Diffraction, 2009 ▶); cell refinement: *CrysAlisPro CCD*; data reduction: *CrysAlisPro RED* (Oxford Diffraction, 2009 ▶); program(s) used to solve structure: *SHELXS97* (Sheldrick, 2008 ▶); program(s) used to refine structure: *SHELXL97* (Sheldrick, 2008 ▶); molecular graphics: *ORTEP-3* (Farrugia, 1997 ▶) and *CAMERON* (Watkin *et al.*, 1993 ▶); software used to prepare material for publication: *WinGX* (Farrugia, 1999 ▶).

## Supplementary Material

Crystal structure: contains datablocks global, I. DOI: 10.1107/S1600536809024829/bt2973sup1.cif

Structure factors: contains datablocks I. DOI: 10.1107/S1600536809024829/bt2973Isup2.hkl

Additional supplementary materials:  crystallographic information; 3D view; checkCIF report

## Figures and Tables

**Table 1:** Hydrogen-bond geometry (Å, °)

*D*—H⋯*A*	*D*—H	H⋯*A*	*D*⋯*A*	*D*—H⋯*A*
C1—H1⋯O1^i^	0.93	2.53	3.416 (3)	159

Symmetry code: (i) 
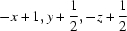
.

**Fig. 1.**
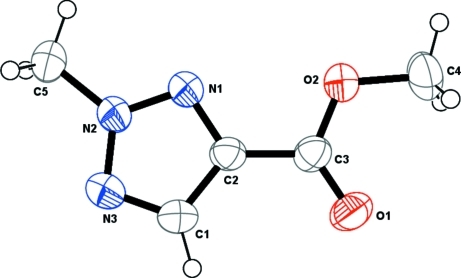
ORTEP diagram of the asymmetric unit of (I) with 50% probability displacement ellipsoids.

## supplementary crystallographic information

### Comment

Triazoles and their derivatives find their application in pharmaceuticals, agrochemicals, dyes, photographic materials, and in corrosion inhibition (Fan & Katritzky, 1996; Dehne,1994; Abu-Orabi *et al.*, 1989). In continuous of our earlier report (Prabakaran *et al.*, 2009), here the crystal structure of the title compound is presented. All non-H atoms lie in a common plane   with maximum deviation of 0.061 (2)° for atom C4. The packing is stabilized by C—H···O hydrogen bonds.

### Experimental

To Methyl 1*H*-1,2,3-triazole-4-carboxylate  (2 g) in dry DMF (15 ml) maintained at 273 K in nitrogen atmosphere, was added K_2CO_3 (1.3 g), metyliodide (ml), the mixture was then stirred at 273 K for 1hr, allowed to warm to room temperature and stirred till completion of reaction, monitored by TLC. The reaction mixture on LCMS analysis showed three isomers well separated with their significant retention time and high purity. Three fractions were identified by mass spectroscopy. The solvent was evaporated under vacuo and the residue was isolated into individual isomers by column chromatography. A portion of the mixture was also analysed by HPLC analysis and also isolated by preparative HPLC techniques. The single crystal of the title compound for X-ray structure anlaysis was obtained from ether solution by slow evaporation.

### Refinement

All the H atoms in  were positioned geometrically and refined using a riding model with C—H bond lenghts of 0.93 Å and 0.96 Å for aromatic and for methyl H atoms respectively and *U*_iso_(H) = 1.2*U*_eq_(C) and *U*_iso_(H) = 1.5*U*_eq_(C_methyl_). The methyl groups were allowed to rotate but not to tip.

### Crystal data

**Table:**

C_5_H_7_N_3_O_2_	*F*(000) = 296
*M**_r_* = 141.14	*D*_x_ = 1.379 Mg m^−^^3^
Monoclinic, *P*2_1_/*c*	Mo *K*α radiation, λ = 0.71073 Å
Hall symbol: -P 2ybc	Cell parameters from 783 reflections
*a* = 3.9482 (10) Å	θ = 2.0–21.4°
*b* = 7.9549 (15) Å	µ = 0.11 mm^−^^1^
*c* = 21.655 (4) Å	*T* = 290 K
β = 92.05 (2)°	Plate, colorless
*V* = 679.7 (2) Å^3^	0.30 × 0.20 × 0.10 mm
*Z* = 4	

### Data collection

**Table:**

Oxford Xcalibur Eos(Nova) CCD detector diffractometer	1262 independent reflections
Radiation source: Enhance (Mo) X-ray Source	910 reflections with *I* > 2σ(*I*)
graphite	*R*_int_ = 0.043
ω scans	θ_max_ = 25.5°, θ_min_ = 3.2°
Absorption correction: multi-scan (*CrysAlis PRO* RED; Oxford Diffraction, 2009)	*h* = −4→4
*T*_min_ = 0.926, *T*_max_ = 0.989	*k* = −9→9
7464 measured reflections	*l* = −26→26

### Refinement

**Table:**

Refinement on *F*^2^	Primary atom site location: structure-invariant direct methods
Least-squares matrix: full	Secondary atom site location: difference Fourier map
*R*[*F*^2^ > 2σ(*F*^2^)] = 0.042	Hydrogen site location: inferred from neighbouring sites
*wR*(*F*^2^) = 0.119	H-atom parameters constrained
*S* = 1.07	*w* = 1/[σ^2^(*F*_o_^2^) + (0.0589*P*)^2^ + 0.0659*P*] where *P* = (*F*_o_^2^ + 2*F*_c_^2^)/3
1262 reflections	(Δ/σ)_max_ < 0.001
93 parameters	Δρ_max_ = 0.19 e Å^−^^3^
0 restraints	Δρ_min_ = −0.16 e Å^−^^3^

### Special details

**Table:**

Geometry. All e.s.d.'s (except the e.s.d. in the dihedral angle between two l.s. planes) are estimated using the full covariance matrix. The cell e.s.d.'s are taken into account individually in the estimation of e.s.d.'s in distances, angles and torsion angles; correlations between e.s.d.'s in cell parameters are only used when they are defined by crystal symmetry. An approximate (isotropic) treatment of cell e.s.d.'s is used for estimating e.s.d.'s involving l.s. planes.

### Fractional atomic coordinates and isotropic or equivalent isotropic displacement parameters (Å^2^)

**Table:**

	*x*	*y*	*z*	*U*_iso_*/*U*_eq_	
N1	0.2453 (4)	0.56849 (18)	0.43005 (7)	0.0442 (4)	
N2	0.3338 (4)	0.71794 (19)	0.45090 (7)	0.0449 (4)	
N3	0.4803 (5)	0.8185 (2)	0.41047 (7)	0.0562 (5)	
O1	0.3755 (4)	0.42664 (18)	0.27655 (6)	0.0620 (5)	
O2	0.1319 (4)	0.29948 (17)	0.35597 (6)	0.0539 (4)	
C1	0.4849 (6)	0.7258 (2)	0.35967 (9)	0.0547 (6)	
H1	0.5707	0.7596	0.3222	0.066*	
C2	0.3408 (5)	0.5700 (2)	0.37131 (8)	0.0407 (5)	
C3	0.2884 (5)	0.4274 (2)	0.32930 (8)	0.0435 (5)	
C4	0.0524 (6)	0.1556 (3)	0.31750 (10)	0.0634 (6)	
H4A	−0.0937	0.1895	0.2834	0.095*	
H4B	−0.0597	0.0720	0.3414	0.095*	
H4C	0.2579	0.1095	0.3022	0.095*	
C5	0.2690 (6)	0.7700 (3)	0.51385 (9)	0.0567 (6)	
H5A	0.1463	0.6831	0.5342	0.085*	
H5B	0.1374	0.8715	0.5130	0.085*	
H5C	0.4805	0.7896	0.5359	0.085*	

### Atomic displacement parameters (Å^2^)

**Table:**

	*U*^11^	*U*^22^	*U*^33^	*U*^12^	*U*^13^	*U*^23^
N1	0.0534 (11)	0.0371 (9)	0.0423 (9)	−0.0034 (7)	0.0041 (7)	0.0000 (7)
N2	0.0581 (11)	0.0348 (9)	0.0419 (9)	−0.0033 (7)	0.0029 (7)	−0.0004 (7)
N3	0.0755 (13)	0.0424 (10)	0.0511 (10)	−0.0100 (9)	0.0069 (9)	0.0050 (8)
O1	0.0904 (12)	0.0553 (9)	0.0413 (8)	0.0058 (8)	0.0164 (7)	0.0007 (6)
O2	0.0715 (10)	0.0457 (8)	0.0450 (8)	−0.0120 (7)	0.0066 (6)	−0.0058 (6)
C1	0.0740 (15)	0.0480 (12)	0.0427 (11)	−0.0060 (10)	0.0109 (10)	0.0065 (9)
C2	0.0454 (11)	0.0381 (10)	0.0388 (10)	0.0017 (8)	0.0034 (8)	0.0050 (8)
C3	0.0502 (12)	0.0422 (11)	0.0382 (10)	0.0072 (9)	0.0018 (8)	0.0038 (8)
C4	0.0752 (16)	0.0449 (12)	0.0699 (15)	−0.0064 (11)	0.0016 (12)	−0.0155 (10)
C5	0.0770 (16)	0.0479 (13)	0.0455 (11)	−0.0022 (10)	0.0067 (10)	−0.0084 (9)

### Geometric parameters (Å, °)

**Table:**

N1—N2	1.315 (2)	C1—H1	0.9300
N1—C2	1.340 (2)	C2—C3	1.464 (3)
N2—N3	1.333 (2)	C4—H4A	0.9600
N2—C5	1.456 (2)	C4—H4B	0.9600
N3—C1	1.325 (2)	C4—H4C	0.9600
O1—C3	1.205 (2)	C5—H5A	0.9600
O2—C3	1.333 (2)	C5—H5B	0.9600
O2—C4	1.444 (2)	C5—H5C	0.9600
C1—C2	1.391 (3)		
			
N2—N1—C2	103.75 (15)	O2—C3—C2	112.31 (16)
N1—N2—N3	115.69 (15)	O2—C4—H4A	109.5
N1—N2—C5	121.67 (15)	O2—C4—H4B	109.5
N3—N2—C5	122.63 (16)	H4A—C4—H4B	109.5
C1—N3—N2	103.33 (16)	O2—C4—H4C	109.5
C3—O2—C4	116.74 (16)	H4A—C4—H4C	109.5
N3—C1—C2	109.13 (17)	H4B—C4—H4C	109.5
N3—C1—H1	125.4	N2—C5—H5A	109.5
C2—C1—H1	125.4	N2—C5—H5B	109.5
N1—C2—C1	108.10 (16)	H5A—C5—H5B	109.5
N1—C2—C3	123.02 (17)	N2—C5—H5C	109.5
C1—C2—C3	128.88 (17)	H5A—C5—H5C	109.5
O1—C3—O2	124.03 (17)	H5B—C5—H5C	109.5
O1—C3—C2	123.65 (18)		
			
C2—N1—N2—N3	0.1 (2)	N3—C1—C2—C3	179.47 (18)
C2—N1—N2—C5	179.01 (17)	C4—O2—C3—O1	−2.7 (3)
N1—N2—N3—C1	0.2 (2)	C4—O2—C3—C2	176.96 (16)
C5—N2—N3—C1	−178.75 (18)	N1—C2—C3—O1	−179.38 (18)
N2—N3—C1—C2	−0.3 (2)	C1—C2—C3—O1	1.7 (3)
N2—N1—C2—C1	−0.3 (2)	N1—C2—C3—O2	1.0 (3)
N2—N1—C2—C3	−179.42 (17)	C1—C2—C3—O2	−177.96 (19)
N3—C1—C2—N1	0.4 (2)		

### Hydrogen-bond geometry (Å, °)

**Table:**

*D*—H···*A*	*D*—H	H···*A*	*D*···*A*	*D*—H···*A*
C1—H1···O1^i^	0.93	2.53	3.416 (3)	159

Symmetry codes: (i) −*x*+1, *y*+1/2, −*z*+1/2.
